# Angiogenic Factors in Women Ten Years after Severe Very Early Onset Preeclampsia

**DOI:** 10.1371/journal.pone.0043637

**Published:** 2012-08-31

**Authors:** Ingrid P. M. Gaugler-Senden, Jouke T. Tamsma, Chris van der Bent, Ron Kusters, Eric A. P. Steegers, Christianne J. M. de Groot

**Affiliations:** 1 Department of Obstetrics and Gynecology, Jeroen Bosch Hospital, ‘s-Hertogenbosch, The Netherlands; 2 Department of Endocrinology and Gastro Intestinal Medicine, Vascular Medicine, Leiden University Medical Center, Leiden, The Netherlands; 3 Department of Clinical Chemistry, Jeroen Bosch Hospital, ‘s-Hertogenbosch, The Netherlands; 4 Department of Obstetrics and Gynecology, Division of Obstetrics and Prenatal Medicine, Erasmus MC, University Medical Center Rotterdam, The Netherlands; 5 Department of Obstetrics and Gynecology, VU Medical Center, Amsterdam, The Netherlands; VU University Medical Center, Netherlands

## Abstract

**Background:**

Women with a history of mainly severe and early onset preeclampsia have an increased risk of future cardiovascular disease. During these complicated pregnancies increased levels of anti-angiogenic factors can be found. We hypothesize that women with a history of severe very early onset preeclampsia still have increased levels of these biomarkers years after this pregnancy, resulting in increased risk for cardiovascular disease.

**Methods:**

Twenty women with severe early onset preeclampsia before 24 weeks' gestation, who delivered between 1993–2003 in a tertiary referral centre and twenty matched controls with uncomplicated pregnancies and healthy term infants, were addressed for participation in the study. Venous plasma samples were analyzed for basic fibroblast growth factor (bFGF), placental growth factor (PLGF), soluble fms-like tyrosine kinase-1 (sFlt-1), vascular endothelial growth factor (VEGF), E- and P-selectin, soluble intercellular adhesion molecule-3 (sICAM-3) and thrombomodulin by ELISA.

**Results:**

Sixteen case subjects and 18 control subjects consented participation. The median time interval index pregnancy to study was 9.4 and 9.7 years for cases and controls, respectively. Median levels for cases-controls (p-value) were not different; bFGF: 17.43–11.11 pg/mL (0.33), sFlt-1: 102.98–101.92 pg/ml (0.84), PLGF: 3.57–4.20 pg/mL (0.38), VEGF: 64.05–45.72 pg/mL (0.73), E-selectin: 5.11–4.68 ng/mL (0.20), P-selectin: 85.35–71.69 ng/mL (0.69), sICAM-3: 0.42–0.63 ng/mL (0.41) and Thrombomodulin: 0.92–0.93 ng/mL (0.59).

**Conclusion:**

There were no differences in angiogenic biomarkers between women with a history of severe early onset preeclampsia versus uncomplicated pregnancy almost 10 years later, suggesting that these angiogenic factors will not contribute to the early detection of women at risk for future cardiovascular disease.

## Introduction

Preeclampsia occurs in 3–5% of pregnancies and is a major cause of both fetal and maternal morbidity and mortality worldwide [1], [2]. The clinical features of the maternal syndrome, hypertension and proteinuria, are based on widespread maternal endothelial dysfunction and microangiopathy [3]. Although, the cause of preeclampsia is unknown, shallow invasion of the trophoblast into the spiral arteries of the placental bed appear to play a key role [4]. Increasing numbers of studies focus on altered expression of angiogenic and anti-angiogenic factors as a result of this impaired cytotrophoblast invasion leading to hypoxia. Current evidence suggests that excess of anti-angiogenic factors mediates symptoms and signs of preeclampsia [5]–[7].

Preeclampsia reflects not only impact on pregnancy itself; epidemiological studies have demonstrated an association between preeclampsia and maternal cardiovascular disease in later life [8]–[11]. Cardiovascular disease (CVD) and preeclampsia, which occurs most often in term pregnancies share many risk factors and pathophysiological abnormalities like hypertension, insulin resistance and increased systemic inflammatory response. Classic risk factors for CVD are hypertension, hyperlipidemia, insulin resistance, and obesity. In the last decade several studies have been published regarding other biomarkers as risk factor for CVD [12]–[18]. These novel factors comprise of angiogenic factors such as vascular endothelial growth factor (VEGF), placental growth factor (PLGF) and basic fibroblast growth factor (bFGF), anti-angiogenic factors such as soluble fms-like tyrosine kinase-1 (sFlt-1) and soluble endoglin (s-Eng) and adhesion molecules such as intercellular adhesion molecule (ICAM), vascular cell adhesion molecule (VCAM), soluble P-selectin and soluble E-selectin. We hypothesize that because of the shared mechanism of hypertensive disorders in pregnancy and CVD later in life, distinct levels of angiogenic and anti angiogenic factors are expressed in women who had preeclampsia and women who had an uncomplicated pregnancy [5], [19]. In addition, women with a history of severe early onset preeclampsia are at the highest risk of CVD and might express distinguished angiogenic risk factors which could be used as markers for secondary preventive measures [20]. Therefore, we studied angiogenic risk factors in a cohort of women with a history of severe preeclampsia with an onset before 24 weeks' gestation (cases) and healthy women with uncomplicated pregnancies (controls). The results of classic cardiovascular risk factors in this rare cohort were published in 2008 [21]. This study showed that on average 5 years (range 4–10 years) after the pregnancy complicated by severe early onset preeclampsia 55% of cases versus 10% of controls had chronic hypertension. There was no difference in BMI, lipid profiles and glucose intolerance between both groups. These findings suggest a more hypertension related vascular etiology rather than a metabolic syndrome origin in severe early onset preeclampsia.

## Methods

### Participants

Twenty women who had been admitted to the University Medical Center Rotterdam between 1993 and 2003, with the diagnosis severe early onset preeclampsia before 24 weeks' gestation and 20 healthy matched control patients after uncomplicated term pregnancies, were addressed for participation in the current study on novel cardiovascular risk factors. Sixteen of 20 cases and 18 of 20 controls consented to participate. Two cases and two controls did not respond to our mailing and two cases refrained from participation as they stated difficulties in blood sampling. All non-participants were of Afro-Caribbean origin. These women did not differ in maternal complications or fetal outcome at time of the index pregnancy from the participating women. The study was approved by the Medical Ethics Committee of the University Medical Center Rotterdam (MEC 2005-185).

Severe preeclampsia was defined as an absolute diastolic blood pressure of ≥110 mm Hg and proteinuria (≥2+ [1 g/l]) on a catheterized specimen on admission, or the occurrence of preeclampsia (blood pressure ≥140 mmHg systolic or ≥90 mmHg diastolic measured on at least two occasions in women normotensive before 20 weeks gestation and proteinuria ≥300 mg/24 h (or ≥2+ on dipstick of voided specimen) in combination with eclampsia or HELLP syndrome. HELLP (hemolysis, elevated liver enzymes, and low platelets) was defined as thrombocytes <100×10^9^/l, and both ASAT (aspartate aminotransferase) and ALAT (alanine aminotransferase) >70 U/l and lactate dehydrogenase >600 U/L.

### Data collection

Venous blood samples were obtained in EDTA collection tubes. A standard laboratory procedure was implemented for the centrifugation, aliquoting and storage of samples at −70°C until assay. Plasma was assayed for bFGF, PLGF, sFlt-1, VEGF, E- and P-selectin, sICAM-3 and thrombomodulin by ELISA (MSD® 96-Well MULTI-SPOT® Vascular Injury Panel I Assay and MSD® MULTI-SPOT® Human Growth Factor I Assay). Samples below detection level were excluded from analyses (bFGF: 2 cases and 1 control, PLGF in 7 cases and 10 controls). All assays were performed by a single investigator (C.B.) and were analyzed blindly and in duplicate. The inter and intra assay coefficients of variation were less than 10%.

### Statistical analyses

Continuous variables are expressed as medians with ranges. General characteristics were compared between groups using independent T-test and χ2 statistics test where appropriate.

The statistical package used was SPSS 18.0 (Chicago, Illinois). A p-value of <0.05 was considered significant.

## Results

General characteristics of participants are demonstrated in Table 1. As expected no differences between women who had preeclampsia and uncomplicated pregnancies were found for maternal age, parity, race and time since index pregnancy (9.4 years vs. 9.7 years in cases and controls, respectively). With regard to the index pregnancy cases delivered significantly earlier and delivered neonates with significantly lower birth weights. In the current study, almost ten years after the index pregnancy, six women (38%) used antihypertensive medication of which five patients had chronic hypertension at time of their (index) pregnancy complicated with severe, early onset preeclampsia before 24 weeks' gestation. None of the controls used anti hypertensive medication.

**Table 1:** General characteristics of women with severe, very early onset preeclampsia and controls.

	Cases	Controls	p value
	n = 16	n = 18	
**Index pregnancy data**			
Age, years	32.5 (29.1–36.2)	31.7 (28.2–35.2)	0.77
Parity			0.37
*Nulliparous, n (%)*	10 (63)	11 (61)	
Race, n (%)			0.50
*Caucasian*	12 (75)	12 (67)	
*African (-Caribbean)*	2 (12.5)	4 (22)	
*Asian*	2 (12.5)	2 (11)	
Gestational age at delivery, weeks	22.8 (22.3–23.3)	40.2 (38.6–41.1)	<0.001
Birth weight, grams	520 (415–600)	3373 (2873–3648)	<0.001
**Data at present study**			
Age, years	42.9 (38.8–45.1)	41.6 (38.8–45.7)	0.86
Time since index pregnancy, years	9.4 (9.2–10.3)	9.7 (9.3–10.9)	0.81
Antihypertensive medication, n (%)	6 (38)	0	0.01

Data are expressed as median (interquartile range).

The results of angiogenic risk factors are depicted in table 2. None of the angiogenic factors were significantly different between cases and controls.

**Table 2:** Angiogenic risk factors in women with severe, very early onset preeclampsia and controls.

	Cases	Controls	p-value
	N = 16	N = 18	
bFGF, pg/mL	17.43 (6.11–35.23)	11.11 (4.16–31.47)	0.33
sFLT-1, pg/mL	102.98 (89.43–109.64)	101.92 (77.63–123.62)	0.84
VEGF, pg/mL	64.05 (50.45–101.87)	45.72 (32.39–78.76)	0.73
E-selectin, ng/mL	5.11 (2.89–7.74)	4.68 (3.27–7.82)	0.20
P-selectin, ng/mL	85.35 (41.94–102.20)	71.69 (58.13–108.00)	0.69
s-ICAM-3, ng/mL	0.42 (0.42–0.73)	0.63 (0.43–0.73)	0.41
Thrombomodulin, ng/mL	0.92 (0.72–1.23)	0.93 (0.75–1.09)	0.59
bFGF, pg/mL	17.43 (6.11–35.23)	11.11 (4.16–31.47)	0.33

Data are expressed in median (interquartile range).

## Discussion

We found no differences in angiogenic risk factors between women with a history of severe preeclampsia with an onset before 24 weeks' gestation (cases) and healthy women who had an uncomplicated pregnancy (controls) ten years after their pregnancy. Although, hypertension was found more prevalent amongst cases, antihypertensive drug use was not associated with higher levels of angiogenic biomarkers. Our conclusion has to be taken with some caution as the sample size of the cohort is small. However, the cohort comprises of women with extreme early and severe disease and a substantial number of women have chronic hypertension, so one would expect this particular group of women to present higher levels of these angiogenic biomarkers. Data on dietary salt intake, which influences levels of angiogenic factors, were not available in our study. Regarding the effect of phases of menstrual cycle at time of blood sampling there was no difference between both groups. Among the cases 8 women had spontaneous cycles and 8 women used oral contraceptives. In four women (50%) with spontaneous cycles blood samples were taken in the follicular phase. Among 18 controls 12 women had spontaneous cycles, 4 women used oral contraceptives and 2 women were post menopausal. Preovulatory blood sampling occurred in 5 women (42%).

Angiogenic and anti-angiogenic factors have been studied during normal and preeclamptic pregnancies. Increased levels of anti-angiogenic factors (sFlt-1 and s-Eng) and lower levels of angiogenic factors (VEGF and PLGF) have been described in women with severe and/or early onset preeclampsia [5], [22]–[26]. Literature on these factors focuses mainly on discriminatory and predictive abilities for preeclampsia as altered levels of angiogenic factors are detectable as early as in the first trimester [19], [27]–[29]. In contrast, knowledge of angiogenic factors after preeclamptic pregnancies is scarce. Noori et al. [30] studied angiogenic factors and maternal vascular function prospectively in 159 women during pregnancy until 12 weeks post partum. Levels of, PLGF, sFlt-1 and s-Eng showed a 50-fold, 25-fold and 2.5-fold fall respectively, from their highest level in the third trimester to the lowest level 12 weeks post partum in women with uncomplicated pregnancies. However, post partum PLGF levels in patients with preeclampsia and gestational hypertension were significantly higher compared to women who had been normotensive during pregnancy. The fall of 3^rd^ trimester to postpartum PLGF levels in normotensive women is higher compared to women with hypertensive pregnancies. They suggest that persistence of increased levels of PLGF is responsible for the increased risk of cardiovascular disease in later life. We were unable to trace any literature with longer follow up of PLGF after pregnancies complicated by preeclampsia. Placental growth factor is expressed not only in placental cells but also in many non-placental cells including endothelial cells. PLGF promotes angiogenesis and is of major importance in pregnancy, but also stimulates atherosclerotic intimal thickening [31]. Elevated PLGF levels were associated with an increased risk of coronary heart disease in the Nurses' Health Study [12] more then ten years after a baseline test in asymptomatic women. Data regarding their pregnancies were not reported. In our study PLGF was below the detection limit of the assay in almost half of the samples. It may be speculated that the younger age and premenopausal state of most of our participants explain the low levels.

Sattar et al. studied angiogenic risk factors in women with a history of preeclampsia, 15–25 years after the index pregnancy [20]. Forty women with preeclampsia and matched controls were analyzed for ICAM-1, VCAM-1 and E-selectin. In this study the median gestational age at delivery was near term, in contrast to our study, in which all patients had *severe* preeclampsia and delivery at 23 weeks. They found an increased concentration of ICAM-1, which is an adhesion molecule involved in monocyte attachment and transformation to macrophages in the vascular wall and appears an independent predictor of coronary heart disease [13]. To our knowledge, there are no other studies on long term follow up after preeclampsia with respect to bFGF, VEGF, soluble P- and E-selectin and thrombomodulin. However, at shorter follow up after preeclampsia Wolf at al [32] found increased levels of sFlt-1 in 29 women at 18 months postpartum and Saxena et al [33] found increased sFlt-1 levels after angiotensin II infusion in 10 women on average 13 months postpartum. In both studies time of onset of preeclampsia is not stated. Similar to our results Yinon et al [34] could not demonstrate differences in angiogenic factors (VEGF, sFlt-1, PLGF and s-End) in 24 women with both early onset (n = 15) and late onset preeclampsia (n = 9) at 6–24 months postpartum. VEGF and sFlt-1 is described to be associated with cardiovascular disease [14], [15] and sFlt-1 correlates with severity of disease [16]. Although, these findings are not consistent, VEGF seems not to be an independent risk factor for cardiovascular disease when adjusted for gender, age, smoking and diabetes [17]. With respect to adhesion molecules E- and P-selectin an association with coronary heart disease seems likely [18], however measurements in a prospective study and meta analyses add no further predictive information to that provided by more established risk factors [35].

It has previously been hypothesized that persistent endothelial dysfunction caused by damaged endothelium during preeclampsia, possibly secondary to exposure to anti-angiogenic factors, may be responsible for these adverse long term cardiovascular outcomes. However, the results of our study on angiogenic risk factors, which are most likely associated with severe and early preeclampsia, do not suggest such a pathophysiological mechanism at long term follow up in this particular subset of patients. We speculate that the imbalance of angiogenic factors as found in preeclamptic pregnancies arise at the moment when the cardiovascular system is stressed by that pregnancy. Similar to the phenomenon of recovery of hypertension and proteinuria after preeclampsia, which may take up to two years in some women [36], the angiogenic imbalance seems to recover as well. In later life the highest associations of angiogenic factors with cardiovascular disease seem to be found in patients with manifest cardiovascular disease, and may be a reflection of metabolic alterations, endothelial activation and low grade inflammation known to be present in that situation.

In summary, we found no differences in angiogenic biomarkers between women with a history of severe preeclampsia and control women ten years after pregnancy. These findings suggest that these angiogenic biomarkers are not useful for risk assessment for future cardiovascular disease in these women.
